# Supplementary material for: Modification of Sugar Profile and Ripening in Atemoya (Annona × atemoya Mabb.) Fruits through Copper Hydroxide Application
Source: Plants (Basel). 2023 Feb 8;12(4):768. doi: 10.3390/plants12040768 (PMC9964681; doi:10.3390/plants12040768)
Supplement: Supplementary file 1 [file plants-12-00768-s001.zip › plants-2156385-supplementary.pdf]

Supplementary material

Table S1. F values and Tukey test's significances of all measured variables in fruits of atemoya (*Annona* × *atemoya* Mabb.) cv. Thompson. Botucatu, São Paulo, Brazil, 2021.

Variable	F value	Tukey test's significance
Hydrogen peroxide	44.36	1 %
Lipid peroxidation	70.66	1 %
Trehalose	13.37	1 %
Mannose	17.40	1 %
Glucose	3.77	5 %
Fructose	3.88	5 %
Sucrose	4.12	1 %
Fruit mass	5.10	1 %
Soluble solids	5.89	1 %
pH	3.02	1 %
Titratable acidity	6.74	1 %
Maturation index	29.12	1 %
Peel browning	3.33	1 %

Table S2. Equations to calculate sugar concentration in fruits of atemoya (*Annona* × *atemoya* Mabb.) cv. Thompson. Botucatu, São Paulo, Brazil, 2021.

Substance	Equation	R^2 value
Fructose	$y = 0.0765x + 0.0033$	0.999
Glucose	$y = 0.6074x + 0.0001$	0.999
Mannose	$y = 0.0541x + 0.0094$	0.999
Sucrose	$y = 0.9305x + 0.1282$	0.999
Trehalose	$y = 0.3909x – 0.0000007$	0.999
